# Dextran-tryamine hydrogel maintains position and integrity under simulated loading in a human cadaver knee model

**DOI:** 10.1016/j.ocarto.2024.100492

**Published:** 2024-05-31

**Authors:** G.S. van der Weiden, S.C. Mastbergen, S.K. Both, M. Karperien, F.P. Lafeber, N. van Egmond, R.J.H. Custers

**Affiliations:** a Rheumatology & Clinical Immunology, University Medical Center Utrecht, Utrecht University, Utrecht, the Netherlands; b Developmental BioEngineering, University of Twente, Enschede, the Netherlands; c Department of Orthopedics, University Medical Center Utrecht, Utrecht University, Utrecht, the Netherlands

**Keywords:** Cartilage repair, Knee joint, Cartilage defect, Hydrogel

## Abstract

**Objective:**

This dextran-tyramine hydrogel is a novel cartilage repair technique, filling focal cartilage defects to provide a cell-free scaffold for subsequent cartilage repair. We aim to asses this techniques’ operative feasibility in the knee joint and its ability to maintain position and integrity under expected loading conditions.

**Method:**

Seven fresh-frozen human cadaver legs (age range 55–88) were used to create 30 cartilage defects on the medial and lateral femoral condyles dependent of cartilage quality, starting with 1.0 ​cm^2^; augmenting to 1.5 ​cm^2^ and eventually 2.0 ​cm^2^. The defects were operatively filled with the injectable hydrogel scaffold. The knees were subsequently placed on a continues passive motion machine for 30 ​min of non-load bearing movement, mimicking post-operative rehabilitation. High resolution digital photographs documented the hydrogel scaffold after placement and directly after movement. Three independent observers blinded for the moment compared the photographs on outline attachment, area coverage and hydrogel integrity.

**Results:**

The operative procedure was uncomplicated in all defects, application of the hydrogel was straightforward and comparable to common cartilage repair techniques. No macroscopic iatrogenic damage was observed. The hydrogel scaffold remained predominately unchanged after non-load bearing movement. Outline attachment, area coverage and hydrogel integrity were unaffected in 87%, 93% and 83% of defects respectively. Larger defects appear to be more affected than smaller defects, although not statistically significant (p ​> ​0.05).

**Conclusion:**

The results of this study show operative feasibility of this cell-free hydrogel scaffold for chondral defects of the knee joint. Sustained outline attachment, area coverage and hydrogel integrity were observed after non-load bearing knee movement.

## 1 Introduction

Chondral defects of the knee joint are a major cause of pain and disability in patients [[1], [2], [3]], over time this can progress to osteoarthritis of the knee joint [4] due to limited Intrinsic repair [5,6]. Despite the incidence of chondral defects, many of the established cartilage restoration procedures have substantial drawbacks, including, limited graft availability, donor-site morbidity, inconsistent long-term results and biomechanically inferior repair tissue [[7], [8], [9], [10], [11]]. To repair cartilage tissue with comparable mechanical properties to the native hyaline cartilage, scaffolds offer promise by providing structure and stability for cell growth and proliferation as well as matrix production. Example as matrix-induced autologous chondrocyte implantation (M-ACI) [12] have shown potential, while recent developments focus on cell-free scaffolds. can infiltrate the scaffold. The ideal cell-free scaffold would be biocompatible, biodegradable [13] and provide the circumstances essential for chondrocytes and mesenchymal stem cells freely present in the knee joint to infiltrate, differentiate and create cartilage matrix. Due to their resemblance to the cartilaginous matrix, hydrogels consisting of natural polymers are therefore suitable options [[14], [15], [16]].

This dextran-tyramine injectable Hydrogel implant has been developed for treatment of localized cartilage defects in the knee joint. The hydrogel consists of natural polysaccharide-conjugates (dextran-tyramine (Dex-TA) and hyaluronic acid–tyramine (HA-TA)) that crosslink under the influence of horseradish peroxidase (HRP) and non-toxic concentrations of hydrogen peroxide (H_2_O_2_) into a stable hydrogel network, that adheres to the surrounding cartilage and bone. The hydrogel facilitates ingrowth of chondrocytes and mesenchymal stem cells [17,18]. The hydrogel can withstand forces between 15 and 20 ​kPa, which corresponds with the forces chondrocytes are exposed to in the cartilage matrix [19]. The hydrogel treatment was compared to microfracture treatment in an equine chondral defect model with a follow-up of 7 months. At final follow-up the hydrogel treated joints showed significantly better histological International Cartilage Regeneration & Joint Preservation Society (ICRS)-II scores [20] compared to the golden standard micro fracture (72% ​± ​7%; vs. 48% ​± ​10% respectively; mean ​± ​SD, where 100% denotes normal cartilage; p ​= ​0.0007) [21,22].

In the development of novel approaches in cartilage regeneration therapy, the clinical handling and application is commonly overlooked, whilst this is likely to influence treatment outcome [23]. Fixation of scaffolds can be achieved by using fibrin glue, suturing, press-fitting, subchondral bone anchoring or a combination of various techniques. These methods, however, have specific drawbacks. Fibrin glue is good for early fixation, though fixation is usually short lasting and not as strong as other techniques. Suturing, subchondral bone anchoring and press-fitting can damage the scaffolds and surrounding cartilage tissue [[24], [25], [26]]. With this study we aim to test the operative feasibility and fixation of this novel cartilage repair technique in the knee joint and improve upon the surgical technique. We hypothesize that the hydrogel will show early stability and maintain its position and integrity under expected loading conditions.

## 2 Material and methods

### 2.1 Injectable hydrogel

The Injectable Hydrogel (CartRevive, Hy2Care) is recently developed and consists of a two-component injectable and bioresorbable hydrogel intended for treatment of cartilage defects in the knee. The hydrogel consists of tyramine conjugates of naturally occurring polymers such as dextran (Dex-TA) and hyaluronic acid (HA-TA), which crosslinks under the influence of HRP and non-toxic concentrations of H_2_O_2_ [18,19]. The cartilage defect needs to be primed prior to placement of the hydrogel.

Materials were stored at a temperature between 2 and 8 ​°C. The hydrogel is prepared a maximum 30 ​min before placement into the cartilage defect. The polymer solution (1.2 ​ml of 11 ​wt% HA-TA/Dex-TA in Phosphate-buffered saline (PBS)) is combined and mixed with HRP (0.6 ​ml of 30 units/ml HRP in PBS), forming the primer and the first component of the hydrogel. The hydrogen peroxide (1.0 ​ml of 3 ​wt% H_2_O_2_) is diluted with saline (5.0 ​ml of 0.9% Sodium Chloride) and subsequently combined and mixed with the polymer solution (0.8 ​ml of 11 ​wt% HA-TA/Dex-TA in PBS), forming the second component of the hydrogel.

**Fig. 1:**
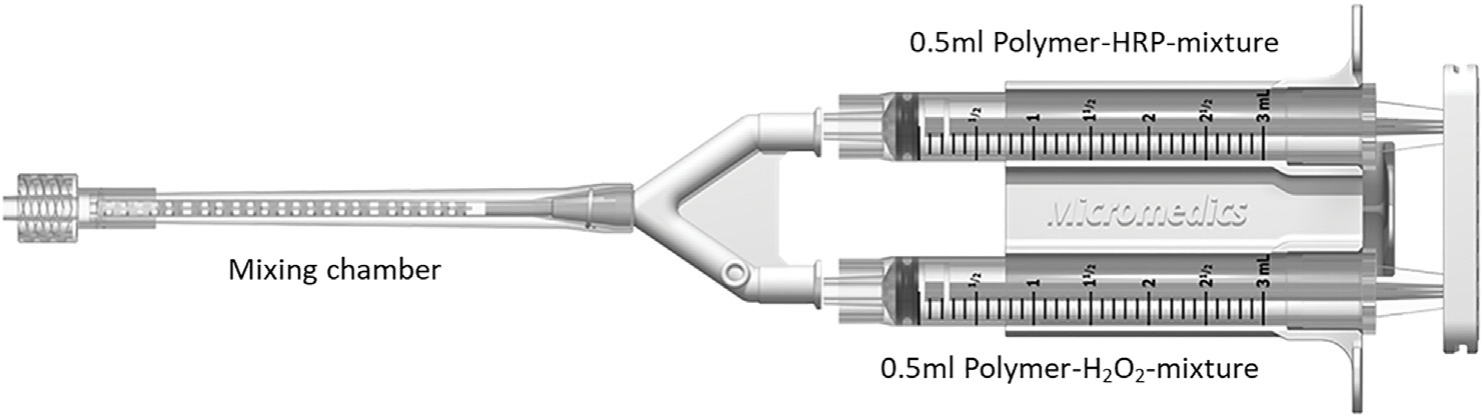
Blending syringe, consisting of mixing chamber, Polymer-horseradish peroxidase (HRP)-mixture and Polymer-hydrogen peroxide (H_2_O_2_)-mixture.

The primer (Maximum 0.5 ​ml of 8.9 ​wt% HA-TA/Dex-TA, 6U/ml HRP in PBS) is placed into the defect using a conventional 1 ​ml syringe. The two components of the hydrogel (Maximum 1.0 ​ml of 8.9 ​wt% HA-TA/Dex-TA, 0.05 ​wt% H_2_O_2_, 3U/ml HRP in PBS-saline-solution) are each placed on either side of a blending syringe complemented with a mixing chamber (Fig. 1).

### 2.2 Cartilage defects

**Fig. 2:**
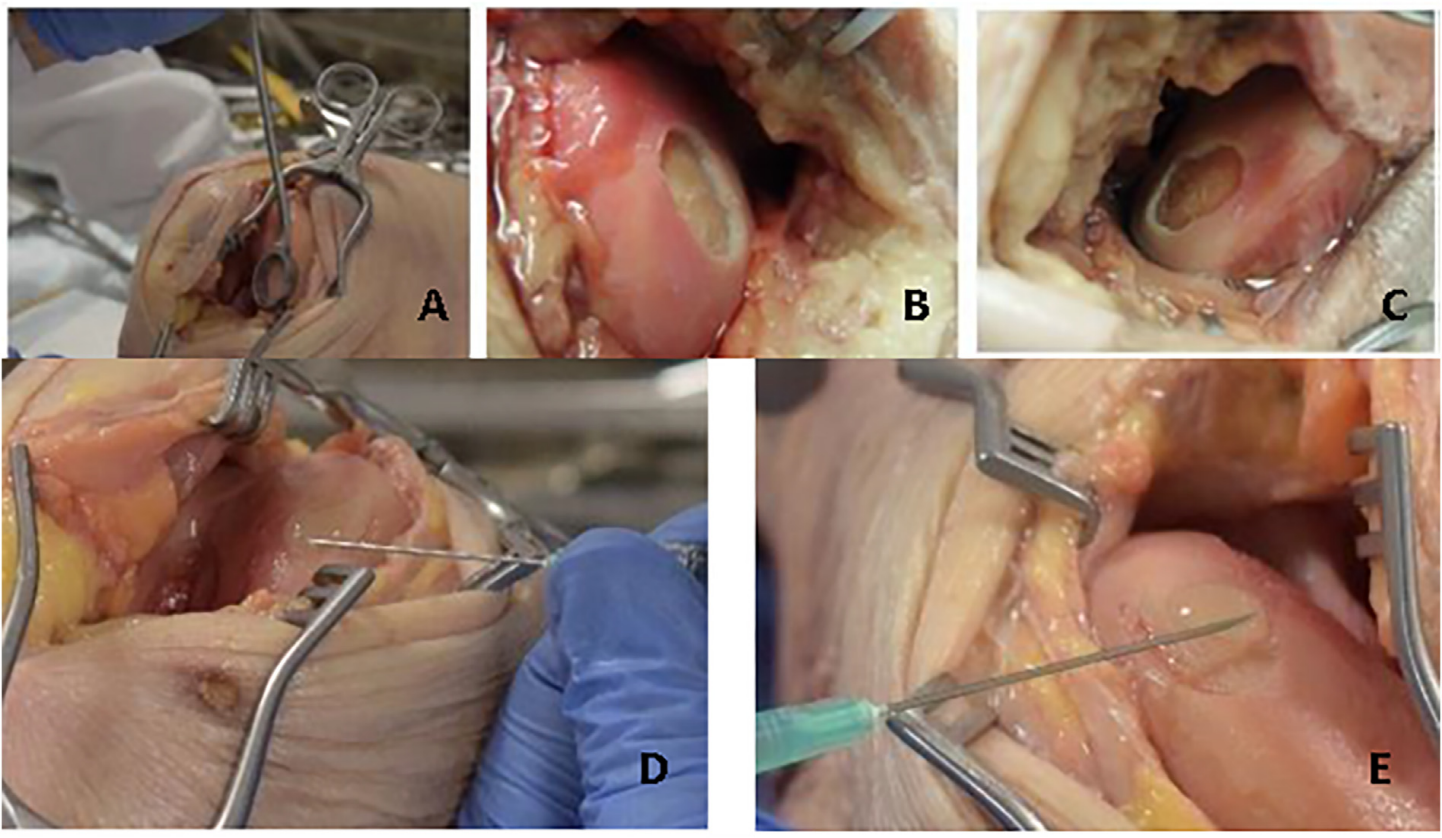
Creating cartilage defects. **A**: Creation of cartilage defect of 2 ​cm^2^ using an oval curette. **B**: Cartilage defect that was created on the Medial femoral condyle of 1.0 ​cm^2^. **C**: Cartilage defect created on the lateral femoral condyle of 2.0 ​cm^2^. **D:** Applying primer in the cartilage defect on the medial, femoral condyle. **E:** Applying hydrogel in the cartilage defect on the medial, femoral condyle.

Seven fresh frozen human cadaver knees, provided by the department of Anatomy of the University Medical Center (UMC) Utrecht, the Netherlands, were thawed by storing it in a refrigerator at 1 ​°C. The donors ranged in age from 55 to 88 years at time of death and consisted of 4 males and 3 females. During the experiment, the pelvis and hip joint were kept intact, and the foot was still attached to preserve mechanics of ligaments and proper movement of joints as much as possible. The legs were subsequently examined for malalignment and manually tested for instability of the knee joint and range of motion (minimum range of motion from 0° extension to 90° flexion). Access to the knee joint was obtained through both a medial and lateral parapatellar incision of the knee joint capsule. Full thickness cartilage defects were made on the weight-bearing section of the medial and lateral femur condyles (MFC and LFC), using a curette and without removal of the calcified cartilage layer (Fig. 2). Fixed templates (NovoCart 3D punch ring by B. Braun; Fig. 2A) were used to create oval sized cartilage defects, starting with a lesion size of 1.0 ​cm^2^. Debris was removed and visually confirmed by the surgeon by the absence of cartilage tissue within the defect. Next, defects were air-dried repeatedly prior to treatment with the hydrogel. After creating the knee cartilage defects, the hydrogel was applied according to manufacturer's instructions. First the primer was applied and 1–2 ​min later the hydrogel was applied. Application of the hydrogel was considered successful if the bottom of the defect was covered and the defect was filled flush with surrounding cartilage. Subsequently, after 1–2 ​min, the knee was closed in layers by stitching and subjected to movement by CPM (see below). The knee was lubricated with the cadaver own knee's synovial fluid, which was sufficient in all cases. After full completion of the CPM protocol and subsequent inspection, the lesion was cleaned and enlarged to 1.5 ​cm^2^, using a fixed template. The new defect was filled with the primer and hydrogel in the same manner and tested using the CPM. The procedure was repeated with an enlargement to 2.0 ​cm^2^ using a fixed template. The procedures followed were in accordance with the ethical standards of the responsible committee on human experimentation (institutional and national) and with the Helsinki Declaration of 1975, as revised in 2000.

### 2.3 CPM

A test set-up using a continues passive motion (CPM) machine (Kinetec® Prima+, provided by Medical sot BV, the Netherlands) was used to simulate movement of the knee joint to mimic the anticipated post-operative regime. At the UMC Utrecht, the Netherlands, there is a strict rehabilitation protocol for patients after treatment of a focal chondral defect in the knee joint. For defects on the femoral condyles this consists of non-weight bearing (maximum 10% of body weight using two crutches) for 6 weeks and restricted movement, starting with passive mobilization guided by pain and reactivity of the knee joint working up to >90° flexion after 6 weeks. Therefore, the unloaded CPM machine was considered a good alternative in this experimental set-up.

**Fig. 3:**
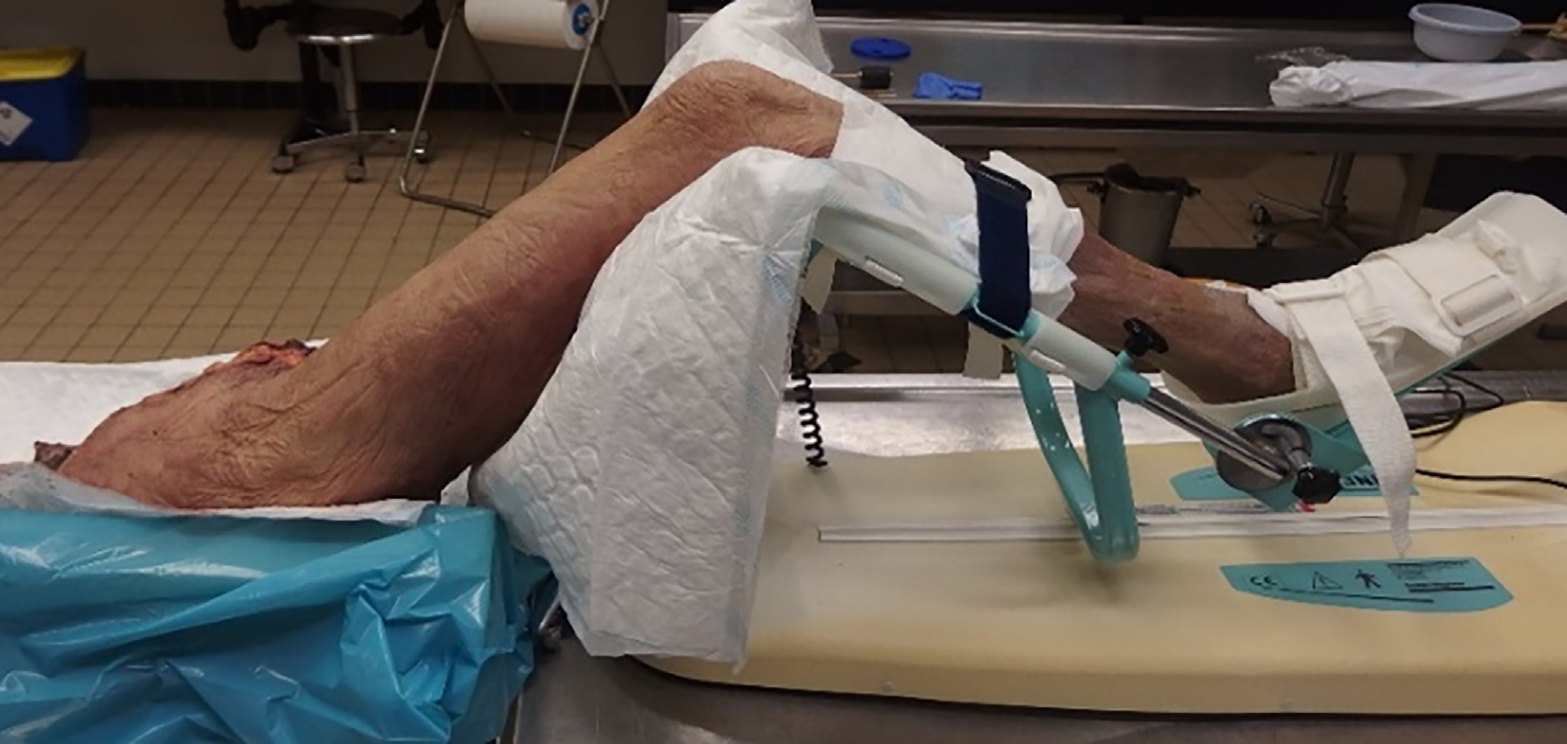
Test set-up with continues passive motion machine was used to simulate movement of the knee joint mimicking the post-operative rehabilitation protocol.

The cadaver leg was placed in the CPM machine for a total of 30 ​min at roughly 1 cycle per minute (Fig. 3), comparable to the maximum permitted motion the days after surgery, as due to pain and reactivity this is usually restricted to this amount for at least 2 weeks. The cadaver leg was secured to the CPM machine using standard straps as might be used in patient care. Two straps for the foot and 1 strap mid-way tibia, ensuring the leg maintains its position in the CPM machine. The machine was set to move from full extension (0°) to 90° flexion, visually verified by the surgeon.

After 30 cycles the joint was opened and the knee joint and the hydrogel scaffold were visually inspected by the surgeon for loss of integrity (damage, crumbling and cracks/tears), dispositioning and/or detachment. High-definition digital photographs (Olympus XZ-10 camera) of the hydrogel scaffold were made for blinded analysis.

### 2.4 Knee simulator

**Fig. 4:**
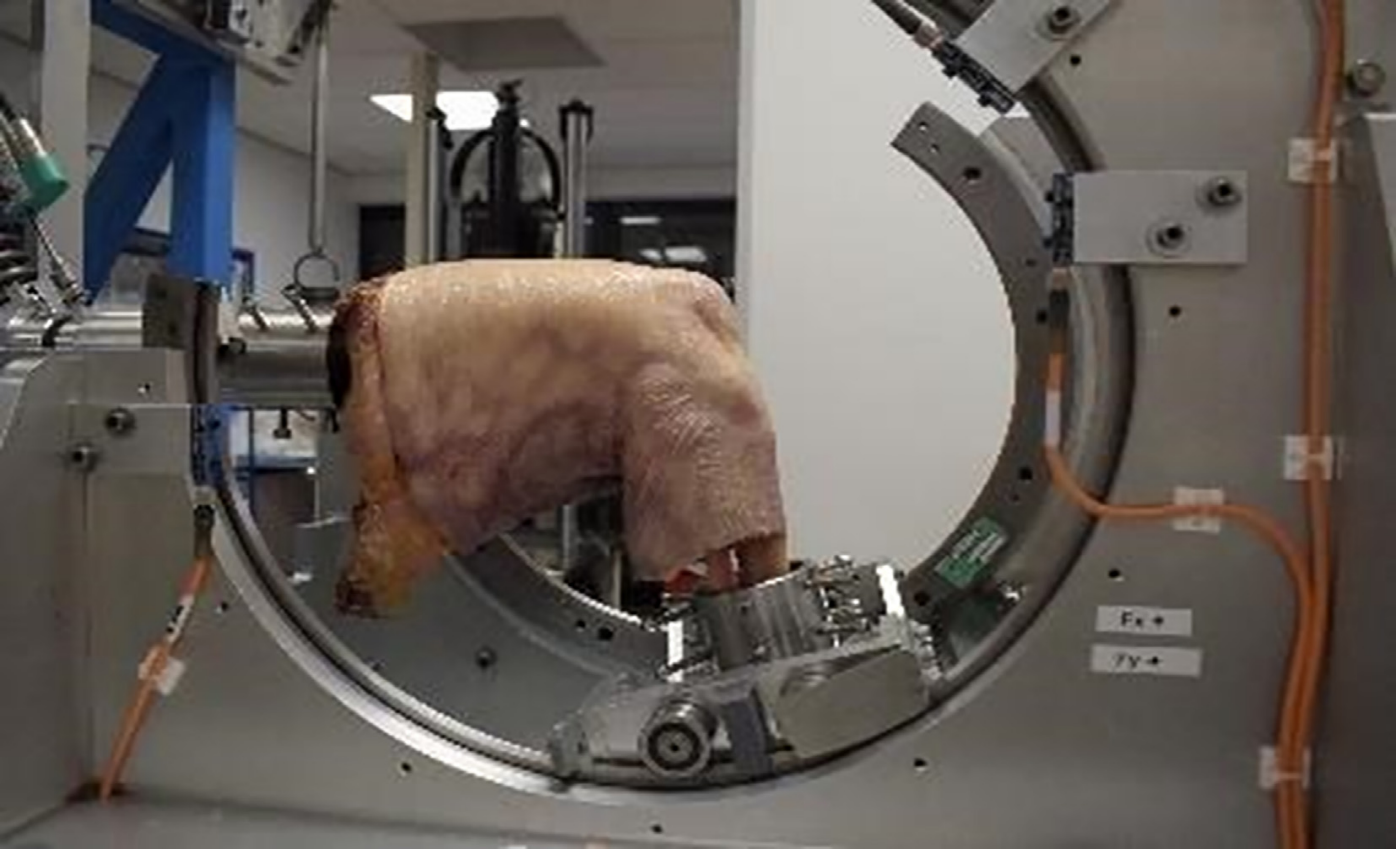
Test set-up with knee simulator used to simulate weight-bearing movement of the knee joint.

Additionally, after completion of the CPM protocol, two donor legs were transported to the orthopedic research laboratory Radboud, Nijmegen, the Netherlands. The knee simulator test setup was used for additional testing to the effect of axial loading on the hydrogel scaffold (Fig. 4). The cadaver leg was modified to fit the test set-up. Femur was cut proximally, whereas the tibia and fibula were cut distally. Both ends were the cemented into a metal vessel to be connected to the test set-up. The knee simulator was subsequently programmed to simulate gait of normal walking conditions. Hereby the knee is cyclically loaded, where the load can be adjusted to preference of the experimental set-up. This type of loading would not be applied within a standard care post-operative regime for cartilage defects, testing was done to assess the loading limitations of the hydrogel.

### 2.5 Scoring system

**Fig. 5:**
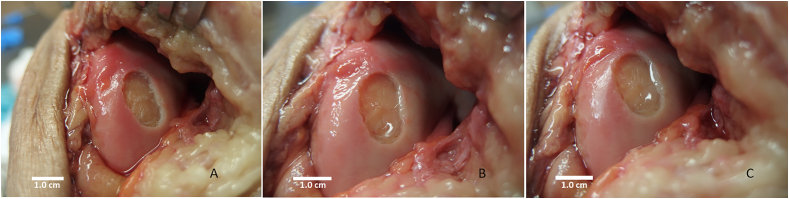
Example of digital photographs provided to independent observers. **A**: Empty cartilage defect of the medial femoral condyle (MFC) size 1.0 ​cm^2^. **B**: Filled cartilage defect directly before movement. **C**: Cartilage defect after completion of continues passive motion (CPM) protocol.

High resolution digital photographs were made of the hydrogel scaffold after placement and directly after completion of the CPM protocol. The digital pictures were compared by three independent observers, blinded for moment, on outline attachment, area coverage and hydrogel integrity. Two pictures were presented per treated defect in random order. One picture showed the defect filled with hydrogel before the CPM usage and the second picture showed the treated defect after completing the CPM protocol. A picture of the empty untreated defect was added as reference to both pictures. Outline attachment was defined as the circumference of the hydrogel in contact with the surrounding cartilage rim. The area coverage as the amount of hydrogel scaffold covering the cartilage defect. The hydrogel integrity was defined as the amount of hydrogel free of shape deformities, fissures or cracks. Observers were asked to compare pictures on each item, scoring the pictures as equal or one of the pictures better than the other (Fig. 5).

### 2.6 Statistical analysis

Interobserver variability was defined as the difference in the measurements between observers. The inter-observer variability were determined by calculating the Intra-Class-Coefficient (ICC) [27] with 95% CIs in a two-way mixed, single-measurement model with absolute agreement by using SPSS (version 23, IBM, Chicago, IL, USA). The ICC values were interpreted according to Fleiss [28] as follows: <0.40 poor, 0.40–0.75 fair to good, and ≥0.75 excellent reproducibility. χ2 tests were used for categorical variables, P-values of ​< ​00.05 were considered statistically significant.

## 3 Results

### 3.1 Chondral defects

A total of thirty full thickness cartilage defects were created, evenly distributed, 10 defects per size (1.0 ​cm^2^, 1.5 ​cm^2^ and 2.0 ​cm^2^). Because of damaged cartilage, due to medial osteoarthritis of the knee in two donors, and iatrogenic damage as a result of staining intents in one donor, a total of seven cadaver knees were used (Table 1).

**Table 1:** Defects created per donor and surgeon observation on displacement of hydrogel scaffold and hydrogel integrity after completion of continues passive motion (CPM) protocol. +**:** indicates no displacement of the hydrogel scaffold/no decline in hydrogel integrity. **-:** indicates displacement of the hydrogel scaffold/decline in hydrogel integrity.

Donor	1.0 ​cm^2^		1.5 ​cm^2^		2.0 ​cm^2^	
	Displacement	Integrity	Displacement	Integrity	Displacement	Integrity
1: Medial	+	+	+	**-**	+	**-**
1: Lateral	+	+	+	+	+	**-**
2: Medial	N.A.^a^	N.A.^a^	N.A.^a^	N.A.^a^	N.A.^a^	N.A.^a^
2: Lateral	+	+	+	+	+	+
3: Medial	N.A.^a^	N.A.^a^	N.A.^a^	N.A.^a^	N.A.^a^	N.A.^a^
3: Lateral	+	+	+	+	+	+
4: Medial	+	+	+	+	+	+
4: Lateral	+	+	+	+	+	+
5: Medial	+	+	+	+	N.A.^b^	N.A.^b^
5: Lateral	+	+	+	+	N.A.^b^	N.A.^b^
6: Medial	+	+	+	+	+	+
6: Lateral	+	+	+	**-**	+	+
7: Medial	N.A.^b^	N.A.^b^	N.A.^b^	N.A.^b^	+	+
7: Lateral	N.A.^b^	N.A.^b^	N.A.^b^	N.A.^b^	+	+
Total	10/10	10/10	10/10	8/10	10/10	8/10

N.A.: Not available.

a Medial side of second and third donor were not usable due to present osteoarthritis.

b in the 5th donor, due to staining with Indian ink, defects sized 2.0 ​cm^2^ were not usable, to have an even distribution, a 7th donor leg was used for defects sized 2.0 ​cm^2^.

There were no complications while applying the gel for all defects. Application of the hydrogel led to consistent filling of the defect with the use of maximum one syringe per defect. During visual inspection after completion of the CPM by operating surgeon, there was no macroscopic damage observed to surrounding or opposing cartilage or surrounding tissue. None of the hydrogel scaffolds were displaced or detached. In 4 out of 30 hydrogel scaffolds, there was an apparent decline in hydrogel integrity. This was observed in 2 defects sized 1.5 ​cm^2^ and 2 defects sized 2.0 ​cm^2^ (Table 1).

### 3.2 Observer scores

Outline attachment of the hydrogel scaffold in the cartilage defect was unaffected following completion of CPM protocol in 26 out of 30 defects. No decline was observed in all 10 hydrogel scaffolds for 1.0 ​cm^2^ sized defects, whereas worsening of outline attachment was seen in 2 out of 10 hydrogel scaffolds of both 1.5 ​cm^2^ and 2.0 ​cm^2^ defects, although this was not statistically significant (Table 2).

**Table 2:** 30 defects scored by three independent blinded observers on outline attachment, area coverage and hydrogel integrity after 30 ​min of knee movement on continues passive motion (CPM) machine.

	1.0 ​cm^2^	1.5 ​cm^2^	2.0 ​cm^2^	Total	ICC
Outline attachment	10/10 (100%)	8/10 (80%)	8/10 (80%)	26/30 (87%)	0.68 (95%CI 0.41–0.83)
Area coverage	10/10 (100%)	9/10 (90%)	9/10 (90%)	28/30 (93%)	0.67 (95%CI 0.39–0.83)
Hydrogel integrity	10/10 (100%)	8/10 (80%)	7/10 (70%)	25/30 (83%)	0.55 (95%CI 0.17–0.77)

**Outline attachment:** Circumference that is in contact with the surrounding cartilage rim. **Area coverage:** Total cartilage defect that is covered by scaffold. **Hydrogel integrity:** Hydrogel free of shape deformities, fissures or cracks. **ICC:** inter-observer variability of three independent blinded observers on outline attachment, area coverage and hydrogel integrity.

Area coverage of the hydrogel scaffold in the cartilage defect showed similar results as found in outline attachment. In 28 out of 30 defects the area coverage of the hydrogel scaffold remained unchanged following completion of the CPM protocol. In the 1.0 ​cm^2^ defects, none of the hydrogel scaffolds area coverage deteriorated, whereas 1 out of 10 hydrogel scaffolds for both 1.5 ​cm^2^ and 2.0 ​cm^2^ defects showed worsening (P ​> ​0.05) (Table 2).

The integrity of the hydrogel scaffold was unaffected in 25 out of 30 defects after completion of the CPM protocol. Here the integrity remained equal for all 1.0 ​cm^2^ defects, in 2 out of 10 1.5 ​cm^2^ defects and in 3 out of 10 2.0 ​cm^2^ defects the integrity of the hydrogel scaffold worsened. This was, however, not statistically significant (Table 2).

The ICC inter-observer values for the three observers were 0.68, 0.67 and 0.55 for outline attachment, area coverage and hydrogel integrity, respectively (Table 2).

### 3.3 Knee simulator

Two cadaver knees with defects of 2.0 ​cm^2^ on both the medial and lateral femur condyles were reused and subjected to movement in the knee simulator after placement of the hydrogel scaffold. In both occasions the knee simulator was programmed to start with low weight bearing gait simulation, which would be the first step in rehabilitation after non-weight bearing motion. For the movement to be reasonably stable, the starting weight was 200 ​N, which roughly translates to 20 ​kg. With a stride frequency of 58.8 strides per minute, which corresponds to slow walking [29]. After 30 cycles the knee was reopened for inspection, after which the axial load could be increased. This resulted in macroscopically visible displacement and destruction of the hydrogel in both cases once the 200 ​N of axial load was passed.

## 4 Discussion

The hydrogel scaffold was able to maintain its position and integrity satisfactorily after minimal non-weight bearing movement on the CPM machine. Direct visual inspection by operating surgeon showed no displacement or detachment of the hydrogel scaffolds after motion. Although visual loss of integrity in 4 out of 30 hydrogel scaffolds, two 1.5 ​cm^2^ and two 2.0 ​cm^2^ sized defects, was observed. Independent observer assessment showed outline attachment, area coverage and integrity of the hydrogel scaffold was maintained after motion in 87%, 93% and 83% respectively.

Studies reporting on fixation of scaffolds are highly variable in fixation techniques, method for testing stability and endpoints, therefore a direct comparison to existing literature is not possible. Bekkers et al. described fixation of a scaffold with fibrin glue, transosseous fixation, biodegradable pin fixation and continuous cartilage sutures, showing better scaffold integrity for fibrin glue compared to trans osseous fixation cartilage sutures, though endpoint fixation was highest for the cartilage sutures, whereas fibrin flue showed weak final fixation strength. Filardo et al. investigated the fixation of an osteochondral scaffold to solely press-fitting or in combination with fibrin glue, fibrin glue notably improved scaffold fixation regardless of lesion location.

The hydrogel scaffold showed maintained outline attachment, area coverage and integrity in all defects sized 1.0 ​cm^2^. In the larger defects the scaffold appeared to be more affected than in the smaller defects, although not statistically significant (p ​> ​0.05). Due to diminished shouldering from surrounding cartilage, we hypothesize defects sized larger than 2.0 ​cm^2^ are likely exponentially more affected. The Dutch Orthopaedic Association (NOV) distinguishes defects sized <2.0 ​cm^2^ to defects sized >2.0 ​cm^2^, and advice cell-based treatments in defects >2.0 ​cm^2^. We have therefor focused on defects sized smaller defect sizes as the intended clinical implication for the hydrogel is defects sized 0.5 ​cm^2^–2.0 ​cm^2^.

The calcified cartilage layer was not removed when creating the cartilage defects as in our equine model studies we saw direct deterioration of the hydrogel scaffold, with migration of the hydrogel into the subchondral bone, though this could also be caused by macrophages present in the subchondral bone. The calcified cartilage layer facilitates cartilage-bone homeostasis and long-term stability, making it crucial for stable repair [30]. Though others have described removal of the calcified cartilage layer as a critical step in cartilage repair, as it results in improved neocartilage integration [31]. In practice a surgeon's ability to remove or retain the calcified cartilage layer varies greatly [32].

The used experimental set-up of non-load bearing with minimal movement after application of the injectable hydrogel is in line with the local post-operative regimen following cartilage repair procedures [33,34]. This rehabilitation protocol consists of an early protection phase of 6 weeks, with no weight-bearing following the surgery, to protect recovering tissues from excessive loading and shear forces. It is essential that during this period, the hydrogel maintains its position, as it is anticipated that cartilage ingrowth could take place in this phase, potentially improving mechanical properties of the Injectable Hydrogel. Motion on a CPM machine allows for natural movement of the knee joint, including terminal rotation in extension. A 30-min cycle was chosen to adhere to local post-operative guidelines, where passive mobilization is allowed for defects on the femoral condyles guided by pain and reactivity of the knee. Though patient experiences are highly variable, in our experience the first two weeks passive motion is limited to 30 ​min cycles, after which most patients can expand upon the passive mobilization. As well as accounting for possible deterioration of tissue that could take place after longer exposure after defrosting of the donor legs. The postoperative use of CPM is used in most post-operative rehabilitation protocols following cartilage repair procedures [33,34]. Based on the current experiments the applied protocol seems suitable and can be applied in clinical trial setting as well.

Muscle loss, specifically of the quadriceps femurs, after surgical management of chondral defects is of great concern [35]. Early weightbearing and more assertive rehabilitation protocols have been developed to accommodate this concern, which doesn't seem to bring increased risks. Although there is no clear evidence that the timing of weightbearing following surgery affects functional outcome [36]. To assess whether the hydrogel scaffold would resist early weightbearing, additional testing was done on two donor legs. Deterioration was already observed with minimal loading of 200 ​N, which roughly translates to 20 ​kg of loading. However, the machine is not optimized for this type of test. The machine is designed for durance testing of mechanical non-resorbable implants, enduring high loads of at least 800 ​N. As such, the machine has not been calibrated for lower loading. Which led to visible shaking of the machine and loss of terminal rotation in extension. Therefore, deterioration of the hydrogel could possibly be attested to these circumstances. Despite all the flaws of the system, the results might indicate that when weight-bearing of the hydrogel takes place in an early phase, it rapidly deteriorates. Although this is line with our current post-op protocols on weight-bearing after treatment of a cartilage defect, it might be specifically important for the application of this hydrogel scaffold.

Patients can deviate protocol or accidently put weight on the operated leg. With this model we cannot conclude whether this would affect the hydrogel in a negative way. Therefore, until clinical data shows otherwise, adherence to the applicable post-operative movement protocol should be stressed.

The Injectable hydrogel scaffold is transparent and challenging to capture well on a photograph. This makes evaluation of the photographs complex as reflected in the interobserver reliability. This also impedes the use of a more detailed scoring system. To draw reliable conclusions, dichotomous deterioration of outline attachment, area coverage and hydrogel integrity after knee movements was assessed using a consensus-based evaluation. A potential solution to overcome this issue is by adding coloring to the hydrogel, However, any past attempts coloring additives resulted in altering the hydrogel gelating process and therefore makes it unusable. Indian ink application after hydrogel application did not provide adequate coloring.

A limitation inherent to cadaver models is the lack of live tissue. The hydrogel is composed of a mixture of natural polymer conjugates that are mixed intra-operatively and which cross-link in situ by means of a mild enzymatic reaction. After injection of the liquid gel in the cartilage defect, and polymerization, the hydrogel adheres to the surrounding tissue structures with use of a primer. Its principle of operation is (temporary) filling of the chondral defect and providing a scaffold structure, allowing perilesional developing chondrocytes to migrate into and attach to the defect (and gel), eventually proliferating into hyaline-like regenerated cartilage. In fresh frozen donor legs, due to loss of cell vitality, the adherence to surrounding tissue is likely to be inferior to that in live tissue. It is therefore conceivable that higher levels of movement and loading are tolerated by the hydrogel scaffold in vivo. Though final strength of the scaffold in live tissue is achieved after longer in situ time of the scaffold, the early stability of the scaffold is considered crucial to initiate this process.

## 5 Conclusion

The results of this study show surgical feasibility of this cell-free hydrogel scaffold for chondral defects of the knee joint. Sustained outline attachment, area coverage and hydrogel integrity were observed after non-load bearing knee movement. Load bearing knee movement seems detrimental to the hydrogel scaffold in the early phase.

## Author contributions

All authors have made substantial contributions to the conception, design, acquisition of data, analysis, interpretation, drafting of manuscript and final approval of submission.

## Role of funding source

This study was funded by Hy2Care. Hy2Care provided the hydrogel implant only and was not involved in the study design, data analysis and interpretation and manuscript preparation and submission. Hy2care reviewed and provided feedback on the manuscript.

## Declaration of competing interest

Hy2Care is a spin-off company of the University of Twente, the Netherlands, S.K. Both and M. Karperien are both founders of Hy2Care. S.K. Both is currently the Director Applications and M. Karperien is currently the chief scientific officer. G.S. van der Weiden, S.C. Mastbergen, F.P. Lafeber, N. van Egmond and R.J.H. Custers report no conflicts of interest.
