# Supplementary material for: Dextran-tryamine hydrogel maintains position and integrity under simulated loading in a human cadaver knee model
Source: Osteoarthr Cartil Open. 2024 May 31;6(3):100492. doi: 10.1016/j.ocarto.2024.100492 (PMC11211881; doi:10.1016/j.ocarto.2024.100492)
Supplement: Multimedia component 1 [file mmc1.docx]

**Instruction**

A series of 3 pictures will be presented per created defect, the empty defect, the defect filled with hydrogel before CPM and the filled defect after CPM. The empty defect will be recognizable and will be assigned a rounded number, whereas the other images will be randomly assigned with number additive of .1 or .2. Observers will be blinded for which number corresponds with before or after CPM. The images will be compared on outline attachment, area coverage and hydrogel integrity. Scoring is divided into: Image 1 is equal to image 2, Image 1 is superior to image 2 and Image 1 is inferior to image 2.

**Outline attachment:** Circumference that is in contact with the surrounding cartilage rim.
**Area coverage:** Total cartilage defect that is covered by scaffold.
**Hydrogel integrity:** Hydrogel free of shape deformities, fissures or cracks.

| **Outline attachment** | **Area coverage** | **Hydrogel integrity** |
| --- | --- | --- |
| Image 1 is equal to image 2  (1 = 2) | Image 1 is equal to image 2  (1 = 2) | Image 1 is equal to image 2  (1 = 2) |
| Image 1 is superior to image 2  (1 > 2) | Image 1 is superior to image 2  (1 > 2) | Image 1 is superior to image 2  (1 > 2) |
| Image 1 is inferior to image 2  (1 < 2) | Image 1 is inferior to image 2  (1 < 2) | Image 1 is inferior to image 2  (1 < 2) |

| **Number** | **Outline attachment** | **Area coverage** | **Hydrogel integrity** |
| --- | --- | --- | --- |
| Example | 1 = 2 | 1 > 2 | 1 < 2 |
| 1 |  |  |  |
| 2 |  |  |  |
| 3 |  |  |  |
| 4 |  |  |  |
| 5 |  |  |  |
| 6 |  |  |  |
| 7 |  |  |  |
| 8 |  |  |  |
| 9 |  |  |  |
| 10 |  |  |  |
| 11 |  |  |  |
| 12 |  |  |  |
| 13 |  |  |  |
| 14 |  |  |  |
| 15 |  |  |  |
| 16 |  |  |  |
| 17 |  |  |  |
| 18 |  |  |  |
| 19 |  |  |  |
| 20 |  |  |  |
| 21 |  |  |  |
| 22 |  |  |  |
| 23 |  |  |  |
| 24 |  |  |  |
| 25 |  |  |  |
| 26 |  |  |  |
| 27 |  |  |  |
| 28 |  |  |  |
| 29 |  |  |  |
| 30 |  |  |  |
